# A neuroimaging dataset on response inhibition and selective attention in adults and children with and without ADHD

**DOI:** 10.1016/j.dib.2021.107158

**Published:** 2021-05-19

**Authors:** Marisa N. Lytle, Douglas D. Burman, James R. Booth

**Affiliations:** a Department of Psychology, The Pennsylvania State University, University Park, PA, 16802, USA; b Department of Radiology, NorthShore University HealthSystem, Evanston, Illinois, USA; c Department of Psychology and Human Development, Vanderbilt University, Nashville, TN, USA

**Keywords:** fMRI, Inhibition, ADHD, Children, Selective attention, Development

## Abstract

In this article we describe the dataset titled “Response inhibition and selective attention in adults and children with and without ADHD” which is publicly available on OpenNeuro.org. This dataset is comprised of neuroimaging and standardized cognitive assessment scores from 11 adults, 12 children diagnosed with Attention Deficit Hyperactivity Disorder (ADHD) and 15 age matched children without ADHD. Functional Magnetic Resonance Imaging (fMRI) data were collected while participants completed selective attention and response inhibition tasks designed and balanced for within or cross-task comparisons. Previous research utilizing this dataset has yet to explore associations between brain function and cognitive assessment scores or differences in neural processes across stimuli features making this dataset valuable for its future contributions to the field as well as replication of prior findings.

**Specifications Table**

**Table:** 

Subject	Developmental Cognitive Neuroscience
Specific subject area	Neuroimaging of Selective Attention and Response Inhibition in ADHD
Type of data	Tables Images
How data were acquired	1.5 Tesla General Electric (GE) Signa Excite scanner, quadrature birdcage head coil. PsyScope software was used to display tasks and collect behavioral data.
Data format	Raw
Parameters for data collection	Participants had no history of non-English or bilingual background, vision impairment, neurological or psychiatric disorder (including oppositional defiant or conduct disorder), pregnancy or birth complications, significant head injury or loss of consciousness, substance abuse, or contraindications for MRI. Adults and control participants could not be taking medication affecting the central nervous system or have a history of ADHD.
Description of data collection	Data were collected across two to three days. During the first visit, participants were interviewed for eligibility and pediatric participants completed standardized measures of cognitive ability. After the interview and testing, all participants completed practice versions of tasks in a mock MRI setup. Within three days participants returned for a second visit where they completed the fMRI session. For most subjects, fMRI collection was divided into two days where tasks containing a target feature (yellow shapes) were performed during visit two and tasks containing a specific target (red triangle) were performed during visit three.
Data source location	Evanston Hospital and Northwestern University Evanston, IL, USA
Data accessibility	Repository name: OpenNeuro Data identification number: 10.18112/openneuro.ds003500.v1.2.0 Direct URL to data: https://openneuro.org/datasets/ds003500/versions/1.2.0
Related research article	J.R. Booth, D.D. Burman, J.R. Meyer, Z. Lei, B.L. Trommer, N.D. Davenport, W. Li, T.B. Parrish, D.R. Gitelman, M.M. Mesulam, Larger deficits in brain networks for response inhibition than for visual selective attention in attention deficit hyperactivity disorder (ADHD), J. Child Psychol. Psychiatry Allied Discip. 46 (2005) 94–111. https://doi.org/10.1111/j.1469-7610.2004.00337.x. J.R. Booth, D.D. Burman, J.R. Meyer, Z. Lei, B.L. Trommer, N.D. Davenport, W. Li, T.B. Parrish, D.R. Gitelman, M.M. Mesulam, Neural development of selective attention and response inhibition, Neuroimage. 20 (2003) 737–751. https://doi.org/10.1016/S1053-8119(03)00404-X.

## Value of the Data

• The present dataset provides foundational neuroimaging data on attention and inhibition in adults, and children with and without ADHD.
• Research and clinicians interested in development and psychiatric disorders will benefit from this data.
• Multiple tasks and standardized measures allow for the examination of parametric manipulations as well as correlations with cognitive ability.
• Compliance with Brain Imaging Data Structure (BIDS) specifications supports ease of future use.

## 1 Data Description

**Fig. 1:**
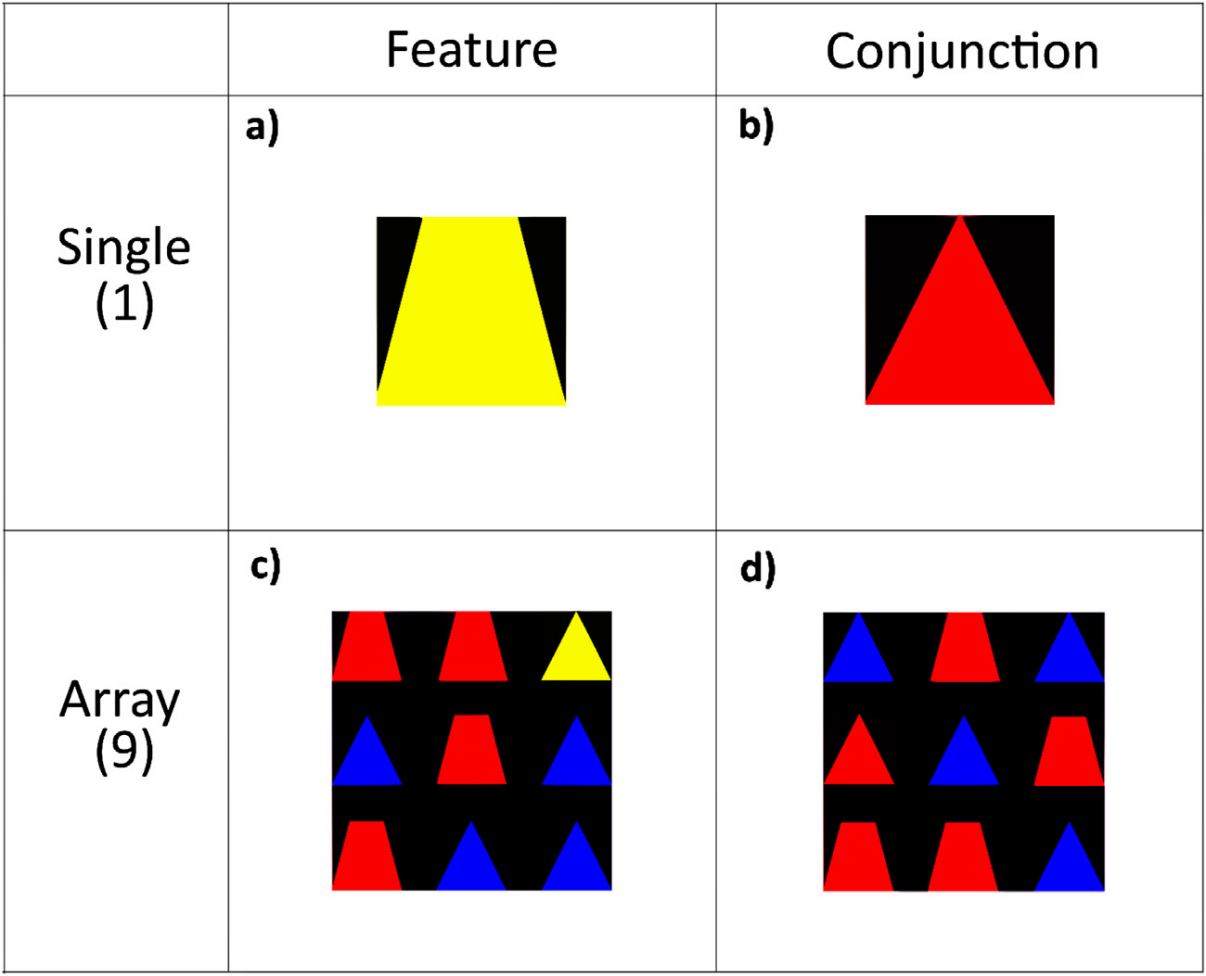
Stimuli by task type. Illustration of trials containing target stimuli in (a) single stimuli feature blocks, (b) single stimuli conjunction blocks, (c) array stimuli feature blocks, and (d) array stimuli conjunction blocks.

Raw neuroimaging data, behavioral task performance, participant demographics, and scores on standardized assessments are available under a CC0 licence on the public neuroimaging repository OpenNeuro.org in the dataset entitled, “Response inhibition and selective attention in adults and children with and without ADHD” [1]. This dataset is organized in accordance with the Brain Imaging Data Structure (BIDS) version 1.4.0 for ease of future reuse as well as compliance with tools that utilize the BIDS structure [2]. The OpenNeuro platform validates BIDS compliance upon upload of the dataset to the repository. The present dataset contains two known warnings which are addressed in the README file accompanying the dataset as well as the De-identification and Quality Control section below. The dataset includes functional MRI images acquired while pediatric participants with ADHD as well as typically developing adults and children completed selective attention and response inhibition tasks designed to allow for cross task comparisons. In addition, this dataset contains block level behavioral data of task performance and structural MRI images for all participants. Scores from standardized assessments of cognitive ability are also included for child participants. The present article includes Fig. 1 which illustrates the stimuli used in the fMRI tasks. This dataset has been used, in part, in two prior publications [3,4]. However, the dataset provides extensive additional data that has yet to be explored including the comparison of cognitive skill to the neural bases of attention and inhibition, as well as the comparison between tasks using feature (yellow shapes) versus conjunction search (red triangle), a difference central to testing theories of selective attention [5].

## 2 Experimental Design, Materials and Methods

### 2.1 Participants

The described dataset includes data collected from 11 adults (9 female, mean age = 25.8 years, range = 20.9-40.9), 12 children with ADHD (4 female, mean age = 11.1 years, range = 9.8-12.3), and 15 children without ADHD (4 female, mean age = 11.3 years, range = 9.4-13.1). Adults and children without ADHD were recruited from the Evanston, Illinois community. Children with ADHD were recruited from pediatric medicine or neurology practices in the Chicago, Illinois area. All children with ADHD had been previously diagnosed with ADHD by a medical professional and were taking medication to treat their ADHD. Children with ADHD were required to withhold taking their ADHD medication for at least 48 h prior to MRI data collection. In addition to diagnosis status, all parents were administered the Disruptive Behavior Rating Scale [6] in order to confirm child's ADHD or non-ADHD group status. Scores provided for each item are included in the phenotype directory at the root level of the dataset in the data file titled *dbrs.tsv*.

As determined in an informal self (adult participants) or parental interview prior to enrollment, all participants had no history of non-English or bilingual background, vision impairment, neurological or psychiatric disorder including oppositional defiant or conduct disorder, pregnancy or birth complications, significant head injury or loss of consciousness, substance abuse, or contraindications for MRI. Additionally, adults and control children could not be taking medication affecting the central nervous system or have a history of ADHD. All procedures were approved by the Institutional Review Board at Northwestern University and Evanston Northwestern Healthcare Research Institute. Informed consent was obtained from participants or parent/guardian(s) including permission for the future release of de-identified data.

### 2.2 Standardized cognitive assessments

After the informal interview to determine eligibility, all child participants were administered a battery of five standardized assessments to quantify cognitive ability. Assessments included: the Comprehensive Test of Phonological Processing (CTOPP) [7], the Peabody Picture Vocabulary Test – Third Edition (PPVT-III) [8], the Wechsler Intelligence Scale for Children –Third Edition (WISC-III) [9], the Woodcock-Johnson III Tests of Achievement (WJ-III) [10], and the Wide Range Achievement Test - Revision 3 (WRAT3) [11]. Composite and standardized scores resulting from testing are provided in the phenotype directory of the dataset and are separated by assessment.

### 2.3 Practice imaging

Following the informal interview and testing, all participants completed a practice MRI session in order to acclimate to the scanner environment. Participants laid supine in a tube-like structure and a button box was placed in their right hand. A computer monitor was positioned 40 cm above the participant's head to view tasks. The experimenter then played digitized versions of sounds the participant may hear when undergoing MRI in order to familiarize the participant with the environment. Once the participant appeared comfortable with the loud noises, they completed a full-length practice version of each experimental task. All tasks were designed and administered with PsyScope presentation software and the PsyScope files for practice tasks are located in the code directory at the root level of the dataset.

### 2.4 MRI acquisition protocol

MR data were acquired using a 1.5 Tesla General Electric (GE) Signa Excite scanner at Evanston Hospital, using a quadrature birdcage head coil. Participants were asked to lie supine in the scanner and their head position was secured using a vacuum pillow (Bionix, Toledo, OH). An optical response box was placed in the participant's right hand to allow them to respond to functional imaging tasks (Lightwave Medical, Burnaby, Canada). Task stimuli were presented through a goggle system attached to the head coil (Avotec, Jensen Beach, FL). At the beginning of each task participants were reminded of the instructions and to keep still.

Structural MRI: T1-weighted SPGR images were collected using the following parameters: TR = 21 ms, TE = 8 ms, matrix size = 256 × 256, slice thickness = 1 mm, number of slices = 124, voxel size = .86 x .86 × 1 mm, flip angle = 20°.

Functional MRI: Blood oxygen level dependent signal (BOLD) was acquired using a T2-weighted susceptibility weighted single-shot echo planar imaging (EPI) and the following parameters: TR = 3000 ms, TE = 40 ms, matrix size = 64 × 64, slice thickness = 4 mm, number of slices = 32, voxel size = 3.437 × 3.437 × 4 mm, flip angle = 90°. Slices were acquired interleaved from bottom to top, odd first. 162 volumes were acquired for each run and the first 6 were removed to allow for equilibration resulting in 156 volumes per run.

### 2.5 Functional imaging tasks

Participants completed two selective attention and four response inhibition tasks in the scanner. In all tasks, participants were instructed to respond as quickly as possible. Half of the tasks required attention to a single feature (any yellow object), whereas the other half required attention to a conjunction of color and shape (red triangle); feature tasks were always performed before conjunction tasks. Except for eight adults who performed all tasks during a single session, feature tasks were performed on the first day in the scanner and conjunction tasks on another day; the selective attention task (either feature or conjunction) was always presented before the corresponding inhibition tasks.

Each task consisted of two alternating conditions. For the selective attention tasks, the first condition consisted of a single stimulus appearing in the center, whereas the second condition consisted of a 3 × 3 array of shapes; in both conditions, the target feature or conjunction appeared on half the trials. At the beginning of each block the participant was reminded of the type of block with an instruction reading ‘One’ or ‘Many’ that lasted 2300 ms followed by 300 ms of blank screen. In the single-stimulus condition, a distractor appeared on the remaining trials, with the same stimulus never appearing in more than three consecutive trials. In the array condition, there were no more than three of the same distractor adjacent on a side, while trials were counterbalanced to include an equal number of targets in each of the nine positions. All stimuli that were not targets were distractors, consisting of blue triangles and red trapezoids for both feature and conjunction tasks. Participants were instructed to respond during attention tasks by pressing the button under their index finger if the target shape was present and press the button under their middle finger if it was absent. The two runs of the selective attention task are titled *Feat19Sel* and *Conj19Sel* for the task containing feature targets and conjunction targets, respectively.

Four response inhibition tasks were used, applying the four stimulus configurations used in the selective attention tasks (see Fig. 1). Inhibition tasks differed from the attention tasks only in their behavioral requirements: the first condition (the “go block”) required a button response to each stimulus appearance, alternating with the second condition (the “no-go block”), which required a button response on every trial *except* when the target appeared. At the beginning of each block the participant was instructed about the type of block with text reading ‘Go’ or ‘Stop’. This instruction lasted 2300 ms, followed by 300 ms of blank screen. Two response inhibition tasks used feature selection for targets (yellow stimulus, one task presenting a single stimulus during a trial and the other presenting an array); the other two inhibition tasks used a conjunction as the target (red triangle, also presented in single-stimulus and array tasks). The four response inhibition tasks are titled: *Feat1Inh, Feat9Inh, Conj1Inh*, and *Conj9Inh*. In these names, “Feat” and “Conj” designate feature or conjunction target types, whereas the 1 or 9 designates the number of shapes in the presented stimuli.

All stimuli used in the described dataset are provided in the stimuli directory at the root level of the dataset. For array stimuli, distractors or target stimuli were presented such that they filled in the vacant position of the array in the provided image file (see bottom of Fig. 1).

For all tasks, stimuli were presented in a series of twelve 18-trial blocks. A stimulus appeared for 1400 ms during a trial, followed by a blank screen during the intertrial interval (ITI). Trials within a block were organized as six 3-trial sets. Each triplet contained one trial with an ITI of 450 ms, another with 600 ms, and a third with an ITI of 750 ms, providing a total duration of 6000 ms for each set. The stimulus configuration and ITI were specified for each triplet, but the order of trials within each set was randomized. This variable duration of ITI was implemented to encourage behavioral responses to actual stimulus appearance, rather than responding at a set pace by anticipating stimulus onset.

Behavioral data are stored alongside imaging files, are titled sub-<sub_ID>_task-<task_name>_ events.tsv, and contain block level data including: onset, duration, intended block type, performed block type, average response time, number of correct responses, number of total errors, number of errors by type, and additional comments. Intended and performed block type were both included because, on occasion, participants would mistakenly perform a go block as a no-go block or vice-versa. In these instances, the number of correct responses and errors was reported based on performed block type. Additionally, participants would sometimes stop responding for periods of three trials or more or would respond in an alternating fashion indicative of off-task behavior. In these instances, the onset and duration of these sets of trials were reported and block type performed was categorized as “NR” to allow for future researchers to segment these data out of their analysis if desired.

### 2.6 De-identification and quality control

Using the freesurfer tool mri_convert [12], imaging data were converted from Analyze format used by early versions of Statistical Parametric Mapping (SPM) to compressed NIfTI format. Facial features were removed from all SPGR images using pydeface (https://github.com/poldracklab/pydeface/) in order to deidentify structural images. Additionally, scanning occurred over the course of one to two days in order to reduce participant fatigue. Date of data acquisition and accompanying behavioral data files are indicated by an acq-{a,b} flag in the data filename. Acquisition dates corresponding to acq-a and acq-b are provided in the participants table at the root level of the dataset and were shifted -365 to 0 days within subjects to protect participant privacy. Years of shifted dates were adjusted to years prior to 1900 to indicate that dates had been de-identified. Shifted participant date of birth is also provided to allow researchers to calculate age at the scanning date of interest.

Neuroimaging data collected from pediatric populations often contains high amounts of movement artifacts. Raw data were inspected for movement using the ArtRepair toolbox [13] with a rejection threshold of greater than 25% of volumes having volume-to-volume movement of greater than one half voxel size (1.7 mm). All data fell below the rejection threshold and thus no data were removed due to movement artifacts.

This dataset is compliant with the BIDS format, however validation of the BIDS format revealed two warnings. One SPGR structural image contained slightly varying imaging parameters which should be considered when reusing the dataset. Furthermore, stimuli files were not referenced in the event files accompanying each functional image due to the block design of the tasks. All stimuli files for all tasks are present in the stimuli directory of the dataset.

## Ethics Statement

Informed consent was obtained from all participants as well as guardians of pediatric participants and all protocols were approved by the Institutional Review Board at Northwestern University 0131-007 and Evanston Northwestern Healthcare Research Institute 99-158. In addition, all identifiable information was removed from the dataset to protect participant privacy.

## CRediT Author Statement

**Marisa N. Lytle:** Data curation, Validation, Writing – original draft; **Douglas D. Burman:** Investigation, Data curtion, Writing – review & editing; **James R. Booth:** Conceptualization, Methodology, Supervision, Funding acquisition, Writing – review & editing.

## Declaration of Competing Interest

The authors declare that they have no known competing financial interests or personal relationships which have or could be perceived to have influenced the work reported in this article.
